# Duration of acute kidney injury and mortality in critically ill patients: a retrospective observational study

**DOI:** 10.1186/1471-2369-14-133

**Published:** 2013-06-27

**Authors:** Seung Seok Han, Sejoong Kim, Shin Young Ahn, Jeonghwan Lee, Dong Ki Kim, Ho Jun Chin, Dong-Wan Chae, Ki Young Na

**Affiliations:** 1 Department of Internal Medicine, Seoul National University College of Medicine, Seoul, South Korea; 2 Department of Internal Medicine, Seoul National University Bundang Hospital, Seongnam-si, South Korea; 3 Department of Internal Medicine, Hallym University Hangang Sacred Heart Hospital, Seoul, South Korea

**Keywords:** Acute kidney injury, Acute renal failure, Duration, Mortality, Survival

## Abstract

**Background:**

The addition of relevant parameters to acute kidney injury (AKI) criteria might allow better prediction of patient mortality than AKI criteria alone. Here, we evaluated whether inclusion of AKI duration could address this issue.

**Methods:**

AKI was defined according to the Kidney Disease: Improving Global Outcomes (KDIGO) guidelines in 2,143 critically ill patients, within 15 days of patient admission. AKI cases were categorized according to tertiles of AKI duration: 1^st^ tertile, 1–2 days; 2^nd^ tertile, 3–5 days; and 3^rd^ tertile, ≥6 days. The hazard ratios (HRs) for overall survival rates in three groups were calculated after adjustment for multiple covariates compared with ICU patients without AKI as the reference group. The predictive ability for mortality was assessed by calculating the area under the curve (AUC) of the receiver operating characteristic curve.

**Results:**

AKI increased the HRs for overall mortality, and the mortality rate increased with AKI duration: the adjusted HRs were 1.99 (1^st^ tertile), 2.67 (2^nd^ tertile), and 2.85 (3^rd^ tertile) compared with the non-AKI group (all *P*s < 0.001). The AUC of the ROC curve for overall mortality based on the AKI duration groups (0.716) was higher than the AUC of AKI staging using the KDIGO guidelines (0.696) (*P* = 0.001). When considering KDIGO stage and AKI duration together, the AUC (0.717) was also significantly higher than that using the KDIGO stage alone (*P* < 0.001).

**Conclusions:**

AKI duration is an additional parameter for the prediction of mortality in critically ill patients. The inclusion of AKI duration could be considered as a refinement of the AKI criteria.

## Background

Acute kidney injury (AKI) is an important field of study in nephrology because AKI leads to long-term kidney sequelae, such as chronic kidney disease (CKD) and end-stage kidney disease [1,2]. Furthermore, AKI affects the fate of other organs and overall mortality [3,4]. Although therapy for AKI has improved in recent years, AKI is still highly prevalent, especially in critically ill patients in the intensive care unit (ICU) [5]. AKI in the ICU has high mortality rates, reaching 80% [6]; these rates have remained largely unchanged despite improvements in therapies [7]. For these reasons, the diagnosis of AKI and its classification according to severity are of major importance for clinicians in managing AKI patients.

Efforts have been made to define and stage AKI cases for use in clinical practice and research. Various criteria for AKI were used in early studies; the first evidence-based consensus, the RIFLE classification, was established by the Acute Dialysis Quality Initiative (ADQI) group [8]. The RIFLE classification defines grades of AKI according to changes in serum creatinine, glomerular filtration rate (GFR), and/or urine output. This RIFLE classification was further refined by the Acute Kidney Injury Network (AKIN) group [9]. The most noticeable change in the AKI criteria defined by AKIN was the inclusion of a smaller change in serum creatinine (≥ 0.3 mg/dL), which may increase the sensitivity of detecting AKI. Recently, Kidney Disease: Improving Global Outcomes (KDIGO) proposed a new set of guidelines for the definition and classification of AKI based on the previous two classifications [10]. Whereas the urine output criterion (UOCr) did not change, some changes in the serum creatinine criterion (CrCr) were made for further clarity and simplicity.

Several epidemiological studies have supported the validity of AKI criteria in predicting mortality. However, identifying only changes in serum creatinine and urine output is not sufficient to improve the reliability of the criteria, thus other factors should be considered. One of the most readily confirmable factors in clinical practice is the duration of AKI. Three previous studies evaluated the association between AKI duration and mortality [11-13], but these results were limited because critically ill patients admitted to the ICU, where the observed negative impact of AKI is the greatest, were not included and because UOCr was not considered. Here, we assessed AKI duration using CrCr and UOCr from the KDIGO guidelines in a large cohort of ICU patients. Using these data, we compared the ability to predict mortality from AKI duration, from the conventional AKI stages, and from AKI duration and stage together to evaluate whether the inclusion of AKI duration could allow better predictions of patient mortality than the conventional AKI criteria alone.

## Methods

### Patients and data collection

The institutional review board at the Seoul National University Bundang Hospital approved the study (no. B-1112-142-103). A total of 2,823 patients were admitted from June 2004 through June 2010 to the ICU at the Seoul National University Bundang Hospital, Gyeonggi-do, Korea. The patients were followed up until December 31, 2010. We excluded patients younger than 18 years old (n = 49) and patients previously diagnosed with end-stage renal disease on dialysis (n = 94). Two patients who remained in hospital at the end of the study were also excluded. Among the study subjects, 9 patients were excluded based on the unavailability of serum creatinine or urine output data. If the patients were admitted more than once to the ICU without AKI, only the first admission was counted as the single case. Consequently, 2,143 patients were reviewed retrospectively using electronic medical records. A standardized data form approved by the institutional review board was used to collect the data.

Clinical parameters, such as age, sex, weight (kg), systolic/diastolic blood pressure, primary diagnosis, underlying CKD, history of malignancy, the need for mechanical ventilation, and the use of vasoactive drugs were recorded. The primary diagnosis was categorized as cardiovascular disease, sepsis, surgical admission, and others. The Acute Physiology and Chronic Health Evaluation (APACHE) ІІ score was used to assess illness severity [14]. Changes in serum creatinine and urine output after ICU admission were measured, and the urine output data were recorded hourly. Additionally, we gathered laboratory blood data such as white blood cell count, hemoglobin, platelet count, blood urea nitrogen, cholesterol, protein, bilirubin, aspartate aminotransferase, alanine aminotransferase, and alkaline phosphatase; there was only about 1% of data missing.

### Definition and classification of acute kidney injury

Definition and staging of AKI were complied with the KDIGO guidelines, which is comprised of CrCr and UOCr (Table 1). Patients were classified as having no AKI or AKI according to the worst stage achieved after admission to the ICU. In the AKI group, the duration of AKI was defined by the number of days that AKI was present from 1 day to the end of AKI (a maximum of 15 days). The end of AKI was determined when it did not conform to the criteria of AKI. For analyses, patients with AKI were divided into three groups according to the tertiles of AKI duration; 1 to 2 days (1^st^ tertile), 3 to 5 days (2^nd^ tertile), and at least 6 days (3^rd^ tertile). Because renal replacement therapy (RRT) can confound AKI duration [15], we categorized patients who underwent RRT into another study group independent of AKI duration. Accordingly, all of the subjects were categorized into the following five groups: the non-AKI group, the 1^st^ tertile group, the 2^nd^ tertile group, the 3^rd^ tertile group, and the RRT group. Recovery from AKI was also examined, and was defined as a return to baseline values of serum creatinine and normal range of urine output within 3 months after AKI event. In-hospital and overall mortalities were selected as mortality outcome; all-cause mortality was considered to be the primary outcome. The mortality data were obtained from hospital records and from the national database of Statistics Korea.

**Table 1:** Definition and staging for acute kidney injury

	**Serum creatinine criterion**	**Urine output criterion**
Stage 1	≥ 0.3 mg/dl increase within 48 hours or 1.5–1.9 times baseline	< 0.5 ml/kg/h for 6–12 hours
Stage 2	2.0–2.9 times baseline	< 0.5 ml/kg/h for ≥ 12 hours
Stage 3	≥ 3.0 times baseline or increase in serum creatinine to ≥ 4.0 mg/dl, or initiation of renl replacement therapy	< 0.3 ml/kg/h for ≥ 24 hours or anuria for ≥ 12 hours

### Statistical analysis

All of the analyses and calculations were performed using SPSS (SPSS version 16.0, IBM, Armonk, NY, USA) and MedCalc software (MedCalc version 11.5.1, Mariakerke, Belgium). The data are presented as means ± standard deviation (SD) for continuous variables and as proportions for categorical variables. Variables with a non-normal distribution are expressed as median (interquartile range (IQR)). The in-hospital survival rates and the overall survival rates which were recorded after the patients had been discharged were calculated using the Kaplan-Meier method. The survival comparison among the groups based on KDIGO stage or AKI duration was performed using the log-rank test. The hazard ratios (HRs) and 95% confidence intervals (CIs) for mortality rates were calculated using the Cox proportional hazard model after adjustment for potential confounders, including age, sex, APACHE ІІ score, primary diagnosis, underlying CKD, history of malignancy, the need for mechanical ventilation, the use of vasoactive drugs, and AKI stage. ICU patients without AKI were used as the reference group. For additional analyses, lengths of hospital stay were compared among the AKI duration groups using the Mann–Whitney *U* test or Kruskal-Wallis test according to the number of groups. The relationship between AKI duration and AKI recovery was evaluated using a Chi-square test. A goodness of fit test was used to evaluate the applicability of the model. The discriminative ability of the criteria to correctly predict mortality was assessed by calculating the area under the curve (AUC) of the receiver operating characteristic (ROC) curve. A comparison of the ROC curves was performed using a method described by DeLong and colleagues [16]. A *P* value of less than 0.05 was considered to be significant.

## Results

### Baseline characteristics

Table 2 shows the baseline characteristics of the study subjects. The mean age was 68 years. All of the subjects were of Asian descent. Most of the patients were admitted to the ICU because of medical problems (n = 2097) rather than surgical problems (n = 46). More specifically, 649 patients (30.3%) were admitted to the ICU because of cardiovascular disease. Sepsis was the cause of admission for 197 patients (4.5%). The mean APACHE ІІ score was 18.1. The median length of stay in hospital was 21 days (IQR, 11 to 43 days). The study subjects were followed for a median duration of 137 days (IQR, 21 to 466 days).

**Table 2:** Baseline characteristics and laboratory findings of the patients at the time of admission to the intensive care unit

	**No AKI (n = 488)**	**1**^**st **^**tertile (n = 552)**	**2**^**nd **^**tertile (n = 497)**	**3**^**rd **^**tertile (n = 437)**	**RRT (n = 169)**
Age (years)	65.0 ± 16.11	66.8 ± 16.49	69.5 ± 15.40	69.7 ± 15.34	66.2 ± 14.30
Male sex (%)	52.5	63.9	59.6	59.3	68.0
Body weight (kg)	58.1 ± 11.61	59.0 ± 13.19	57.1 ± 11.94	57.8 ± 13.45	62.0 ± 11.09
Primary diagnosis					
Cardiovascular disease	39.1	27.4	30.4	27.7	20.1
Sepsis	1.6	3.3	4.8	5.0	14.8
Surgical emergency	3.7	2.5	1.2	1.6	0.6
Others	55.5	66.7	63.6	65.7	64.5
Underlying CKD	5.5	6.9	9.3	9.8	22.5
History of malignancy	16.2	14.7	17.1	19.9	24.3
Need for MV	51.2	62.9	76.7	81.2	78.1
Use of vasoactive drugs	24.2	44.9	59.8	65.7	75.1
SBP (mmHg)	133.1 ± 31.37	132.3 ± 33.58	128.1 ± 35.15	128.4 ± 36.02	118.4 ± 32.54
DBP (mmHg)	71.1 ± 23.61	70.1 ± 26.18	64.8 ± 26.81	63.6 ± 28.42	56.2 ± 25.59
White blood cells (1000/mm^3^)*	9.5 (7.09–12.69)	10.7 (7.40–14.42)	10.9 (7.53 – 15.37)	10.4 (7.45–14.57)	10.1 (7.00–15.67)
Hemoglobin (g/dL)	12.8 ± 2.32	12.7 ± 2.63	12.0 ± 2.58	11.7 ± 2.74	10.8 ± 2.90
Platelet (1000/μL)*	237 (185–294)	217 (163–289)	214 (156–294)	217 (144–292)	177 (88–261)
Blood urea nitrogen (mg/dL)*	15 (11–20)	18 (13–26)	22 (14–34)	22 (14–39)	34 (20–66)
Creatinine (mg/dL)*	0.9 (0.7–1.1)	1.1 (0.8–1.3)	1.2 (0.9–1.7)	1.2 (0.8–1.9)	2.0 (1.1–3.9)
Cholesterol (mg/dL)	170.0 ± 49.48	161.5 ± 56.57	152.4 ± 52.38	153.2 ± 59.00	138.6 ± 50.88
Protein (g/dL)	6.6 ± 0.99	6.5 ± 1.03	6.4 ± 1.04	6.2 ± 1.05	6.1 ± 1.07
Bilirubin (mg/dL)*	0.7 (0.5–1.0)	0.8 (0.6–1.1)	0.8 (0.6–1.2)	0.7 (0.5–1.1)	0.9 (0.5–1.5)
GOT (mg/dL)*	26 (19–39)	31 (20–51)	33 (22–67)	31 (21–58)	36 (22–98)
GPT (mg/dL)*	22 (14–33)	22 (12–41)	22 (14–43)	23 (14–48)	28 (15–65)
Alkaline phosphatase (mg/dL)*	76 (63–99)	83 (63–106)	82 (64–112)	85 (65–118)	84 (66–122)
APACHE ІІ score	13.4 ± 6.51	16.7 ± 7.33	20.7 ± 7.80	20.3 ± 7.86	22.1 ± 8.51
LOS in hospital (days)*	16 (9–35)	21 (11–38)	22 (11–51)	29 (14–54)	13 (7–28)

*Data are expressed as the median (interquartile range).

Duration of acute kidney injury: 1^st^ tertile, 1–2 days; 2^nd^ tertile, 3–5 days; 3^rd^ tertile, ≥ 6 days.

*AKI* Acute kidney injury, *RRT* Renal replacement therapy, *CKD* Chronic kidney disease, *MV* Mechanical ventilation, *SBP* Systolic blood pressure, *DBP* Diastolic blood pressure, *APACHE* Acute Physiology and Chronic Health Evaluation, *LOS* Length of stay.

### Acute kidney injury and its duration

**Figure 1:**
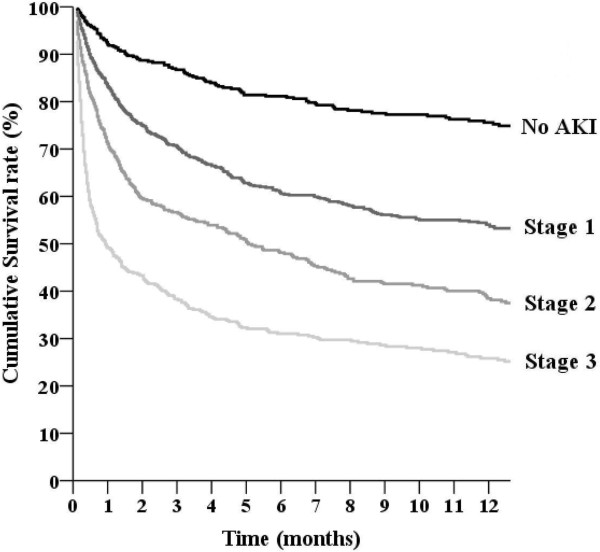
**Overall survival curves of critically ill patients according to the KDIGO (Kidney Disease: Improving Global Outcomes) guidelines for acute kidney injury.** The overall survival rates of the four groups were significantly different (*P* < 0.001 by the log-rank test).

Of the total number of subjects, 1655 (77.2%) were determined to have AKI within 15 days of ICU admission. Each AKI case was diagnosed by CrCr alone (66.4%), UOCr alone (2.9%), or both (30.7%). Among the patients with AKI, the proportion in each AKI stage was as follows: stage 1, 46.5%; stage 2, 27.2%; and stage 3, 26.3%. A total of 169 patients received RRT within 15 days of ICU admission. Throughout the following period, 1,166 (54.4%) of all ICU patients died, and the mortality rate was 169.1 deaths per 100,000 patient-days. The overall mortality rate of the patients with AKI was higher than that of the non-AKI patients (215.3 vs. 64.4 deaths per 100,000 patient-days, *P* < 0.001). Figure 1 shows the overall survival curves when the patients were divided into four groups according to the KDIGO guidelines (*P* < 0.001 by the log-rank test). The HRs (95% CIs) for overall mortality according to AKI stage vs. no AKI were as follows: stage 1, 2.08 (1.71–2.52); stage 2, 3.00 (2.45–3.68); and stage 3, 5.14 (4.21–6.28).

**Figure 2:**
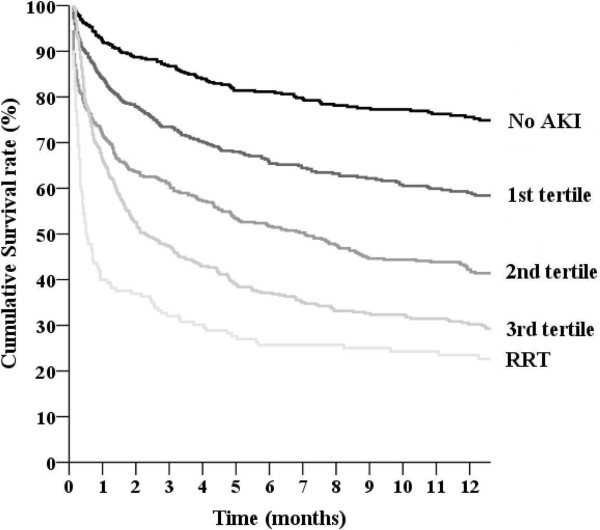
**Overall survival curves of critically ill patients according to the duration of acute kidney injury.** The overall survival rates of the five groups were significantly different (*P* < 0.001 by the log-rank test). Duration of acute kidney injury: 1^st^ tertile, 1–2 days; 2^nd^ tertile, 3–5 days; 3^rd^ tertile, ≥ 6 days. RRT, patients who underwent renal replacement therapy.

**Figure 3:**
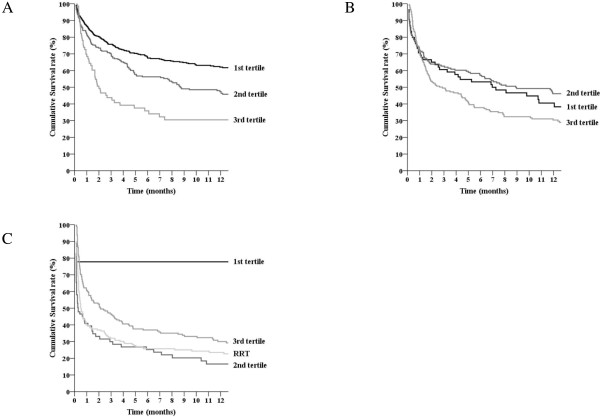
**Overall survival curves according to the duration of acute kidney injury in each KDIGO stage: stage 1 (A), stage 2 (B), and stage 3 (C).** Duration of acute kidney injury: 1^st^ tertile, 1–2 days; 2^nd^ tertile, 3–5 days; 3^rd^ tertile, ≥ 6 days. RRT, patients who underwent renal replacement therapy.

The mean duration of AKI among all AKI patients was 4.5 days (median, 4 days; IQR, 2 to 7 days). The mean duration of AKI when the patients who received RRT were not considered in the analysis was 4.1 days (median, 3 days; IQR, 2 to 6 days). A 1-day increase in AKI duration led to an 11.7% increase in the overall mortality rate (*P* < 0.001). After excluding the patients who received RRT, a 1-day increase in AKI duration was associated with a 13.9% increase in the overall mortality rate (*P* < 0.001). The AKI patients who did not receive RRT were divided into three groups according to the tertiles of AKI duration: 1^st^ tertile (n = 552), 1–2 days; 2^nd^ tertile (n = 497), 3–5 days; and 3^rd^ tertile (n = 437), ≥ 6 days. 92.6% (1^st^ tertile), 98.8% (2^nd^ tertile), and 100% (3^rd^ tertile) of AKI cases could be determined by CrCr alone, whereas 17.0% (1^st^ tertile), 30.2% (2^nd^ tertile), and 38.2% (3^rd^ tertile) of AKI cases could be determined by UOCr alone. The RRT group was defined using CrCr alone (100%), but the RRT group also had AKI cases determined by UOCr alone (85.8%). Figure 2 shows the overall survival curves according to the tertiles of AKI duration; the overall survival rates among the five groups were significantly different (*P* < 0.001 by the log-rank test). The HRs for mortality are shown in Table 3. The mortality rates of the AKI groups were higher than those of the non-AKI group (all *P*s < 0.001). After adjusting for multiple confounding factors including age, sex, APACHE ІІ score, primary diagnosis, underlying CKD, history of malignancy, the need for mechanical ventilation, the use of vasoactive drugs, and AKI stage, the HRs for mortality remained significant (all *P*s < 0.001). Figure 3 shows the overall survival curves for the AKI duration groups for each AKI stage (according to the KDIGO guidelines). The overall survival rates among the AKI groups for each AKI stage were significantly different (all *P* < 0.05 by the log-rank test). The lengths of hospital stay (Table 2) were different among the AKI duration groups (*P* < 0.001); the duration of stay for the non-AKI group was shorter than those of the 1^st^, 2^nd^ and 3^rd^ tertile groups (*P* = 0.004 for 1^st^ tertile and *P*s < 0.001 for 2^nd^ and 3^rd^ tertiles), but was longer than that of the RRT group (*P* = 0.024). The proportions of recovery from AKI were significantly different among the AKI groups: 1^st^ tertile group, 90.4%; 2^nd^ tertile group, 74.4%; 3^rd^ tertile group, 59.0%; RRT group, 21.3% (*P* < 0.001).

**Table 3:** Hazard ratios (95% confidence intervals) for mortality according to the duration of acute kidney injury

	**Unadjusted HR**		Adjusted HR*	
**Duration groups**	**In-hospital mortality**	**Overall mortality**	**In-hospital mortality**	**Overall mortality**
No AKI (n = 488)	1 (Reference)	1 (Reference)	1 (Reference)	1 (Reference)
1^st^ tertile (n = 552)	2.07 (1.41–3.02)	1.77 (1.43–2.18)	3.22 (2.03–5.13)	1.99 (1.48–2.67)
2^nd^ tertile (n = 497)	3.83 (2.69–5.46)	2.97 (2.43–3.63)	4.29 (2.83–6.51)	2.67 (2.04–3.50)
3^rd^ tertile (n = 437)	4.43 (3.12–6.30)	3.85 (3.15–4.70)	3.64 (2.48–5.35)	2.85 (2.24–3.64)
RRT (n = 169)	8.97 (6.18–13.02)	5.73 (4.51–7.27)	5.57 (3.76–8.25)	3.83 (2.96–4.95)

*HR*, Hazard ratio; *AKI*, Acute kidney injury; *RRT*, Renal replacement therapy.

*Adjusted for age, sex, APACHE ІІ score, primary diagnosis, underlying CKD, history of malignancy, the need for mechanical ventilation, the use of vasoactive drugs, and AKI stages.

### Predicting mortality based on the duration of acute kidney injury

The AUC (95% CI) of the ROC curve for overall mortality was 0.696 (0.676–0.715) when AKI was staged according to the KDIGO guidelines, and 0.716 (0.697–0.735) for the AKI duration groups (*P* = 0.001). When AKI duration was considered as a continuous variable (0 to 15 days), the AUC was 0.718 (0.698–0.737); the AUC was 0.711 (0.690–0.731) in the patients who did not receive RRT. The AUC for overall mortality in patients who did not receive RRT was analyzed according to duration as determined by each criterion with the following results: AKI duration using CrCr, 0.670 (0.648–0.690), and AKI duration using UOCr, 0.596 (0.574–0.618). When considering AKI staging according to the KDIGO guidelines and AKI duration together, the AUC for overall mortality (0.717 (0.697–0.736)) was significantly higher than the AUC calculated from the AKI staging (*P* < 0.001). The AUCs for in-hospital mortality were also higher when using AKI staging and AKI duration together (0.751 (0.732–0.769)) than the AUCs calculated using only AKI staging (0.731 (0.712–0.750)) (*P* < 0.001).

## Discussion

AKI worsens the survival of patients with a variety of presenting conditions, especially critically ill patients. Several factors in addition to those currently measured may be involved in the inverse association of AKI with survival. This study shows that AKI duration is an additional predictive parameter for mortality during and after ICU admission. In addition to revealing a strong correlation between AKI duration and mortality, the present data has several strengths. First, we adopted the KDIGO guidelines for AKI, which are the most recently revised guidelines, offering clarity and simplicity in clinical use. Furthermore, we used an hourly collected UOCr. The hourly collection of urine output is essential in staging and defining AKI, but can be difficult and thus has rarely been considered in most previous studies. Therefore, our study results may be a good reference for future studies using the KDIGO guidelines. The second strength of this study is that patients admitted to the ICU were included in our analysis. Two previous studies on AKI duration have used a cohort of patients who underwent cardiac or noncardiac surgery [12,13]. However, those studies did not cover a heterogeneous population of critically ill patients, who are most vulnerable to AKI.

The detection and classification of AKI is important for determining patient prognosis. The RIFLE and AKIN criteria used to define AKI have been shown to be good predictors of patient prognosis [17,18]. The KDIGO guidelines, which represent a combination of RIFLE and AKIN criteria, may also allow the prediction of morbidity and mortality, although few studies using this guideline have been conducted so far. However, the AKI criteria have not yet been fully optimized: some cases of AKI can remain undiagnosed [19,20]; a non-gradual increase in mortality with increasing AKI stage has been shown in some studies [19-22]. These problems can be explained by shortcoming of serum creatinine as an early AKI marker [23], limitation in the use of UOCr [24], and the failure to identify other factors such as the cause of AKI [25]. We were interested primarily in the latter point; among several other predictive factors, we focused on the effect of AKI duration, because this can be easily measured without further extensive tests.

In this study, patients with a long duration of AKI had higher mortality rates and longer hospital stays than patients with a short duration of AKI. The strong correlation between AKI duration and poor outcome can be explained as follows: the more severe and treatment-resistant an AKI case is (e.g., non-recovery case), the longer the duration of AKI become. Three previous studies investigated this issue by dividing patients into groups: transient azotemia (≤ 3 days) and acute tubular necrosis (≥ 4 days or patients with RRT) [11]; short (≤ 2 days), medium (3–6 days), and long (≥ 7 days or patients with RRT) [12,13]. However, we set the patients with RRT apart because patients who received RRT may have cases of AKI that are much more severe than those of other patients and because RRT shortens the duration of AKI compared with other medical treatments [15]. Based on the results thus far, it can be concluded that the effort to recover from AKI is an important consideration, particularly for long-term AKI cases. AKI is not merely a disease of the kidneys, as it can also affect the functioning or failure of other organs [3]. Therefore, early detection and early treatment of AKI are essential to reduce the negative impact of AKI. However, the present study does not underestimate the importance of transient AKI, because short-duration AKI was also found to increase mortality significantly compared to no AKI, and a 1-day increase in AKI duration was associated with a 13.9% increase in overall mortality.

The ability to predict mortality was compared between AKI duration groups and conventional AKI stages (according to KDIGO) by assessing the AUC of the ROC curves. The AUCs of the ROCs indicated that AKI duration had greater ability to predict mortality than the conventional AKI stages. Furthermore, when considering AKI duration and AKI staging together, the ability to predict mortality was superior to the results based on the analysis of AKI staging alone. These results suggest that the AKI duration could be considered as an extra parameter to increase the sensitivity of the KDIGO criteria. Additionally, if AKI staging is not carried out, then AKI duration can be used as a replacement to predict mortality.

Although our results are informative, this study has some limitations. First, the ICU design of the study limits the applicability of our conclusions to other settings despite the abundance and detail of the dataset; the proportion of patients with sepsis as a cause of admission was relatively low. Second, we did not control for the use or indication of treatment for AKI because AKI duration could change depending on the treatment regimen. Further studies addressing these limitations will be necessary in the future.

## Conclusions

In conclusion, AKI duration is a useful parameter for the prediction of mortality in critically ill patients. Comparable predictions of mortality can be made from AKI duration alone. Furthermore, the prediction of mortality can be improved when considering AKI duration in addition to the current AKI criteria. Although the current criteria for detecting and staging AKI are useful in clinical practice and research, the results of the current study should be considered in future refinements of the AKI criteria.

## Competing interests

The authors declare that they have no competing interests.

## Authors’ contributions

SSH and SK designed the study, analyzed the results, interpreted the data, and wrote the manuscript. SYA and JL analyzed the results. DKK and HJC designed the study and interpreted the data. DWC designed the study, analyzed the results, and edited the manuscript. KYN designed the study, analyzed the results, wrote the manuscript, and finally approved the manuscript. All authors read and approved the final manuscript.

## Pre-publication history

The pre-publication history for this paper can be accessed here:

http://www.biomedcentral.com/1471-2369/14/133/prepub
